# Gap Effect on Electric Field Enhancement and Photothermal Conversion in Gold Nanostructures

**DOI:** 10.3390/mi13050801

**Published:** 2022-05-21

**Authors:** Hirotomo Chiba, Kento Kodama, Koki Okada, Yoshiyasu Ichikawa, Masahiro Motosuke

**Affiliations:** 1 Department of Mechanical Engineering, Graduate School of Engineering, Tokyo University of Science, 6-3-1, Niijuku, Katsushika-ku, Tokyo 125-8585, Japan; 4521541@ed.tus.ac.jp (H.C.); 4520530@ed.tus.ac.jp (K.K.); 4522701@ed.tus.ac.jp (K.O.); 2 Department of Mechanical Engineering, Faculty of Engineering, Tokyo University of Science, 6-3-1, Niijuku, Katsushika-ku, Tokyo 125-8585, Japan; yos.ichikawa@rs.tus.ac.jp; 3 Water Frontier Research Center, Research Institute for Science and Technology, Tokyo University of Science, 1-3, Kagurazaka, Shinjuku-ku, Tokyo 162-8601, Japan

**Keywords:** nanomaterial manipulation, localized surface plasmon resonance, gold nanostructure, plasmonic electric field enhancement, photothermal conversion, thermophoresis

## Abstract

Plasmonic optical tweezers and thermophoresis are promising tools for nanomaterial manipulation. When a gold nanostructure is irradiated with laser light, an electric field around the nanostructure is enhanced because of the localized surface plasmon resonance, which increases the optical radiation pressure applied to the nanomaterials. In addition, a temperature gradient is also generated by the photothermal conversion, and thermophoretic force is then generated. This study numerically evaluated the electric and temperature fields induced by the localized surface plasmon resonance between two gold nanostructures. Here, we focused on the effect of the gap width between nanostructures on the optical radiation pressure and thermophoretic force. The simulation results show that the electric field is locally enhanced according to the gap width, but the effect on the temperature rise due to the photothermal heating is small. This fact suggests that the gap effect between the nanostructures is particularly dominant in nanomanipulation using optical force, whereas it has little effect in nanomanipulation using thermophoresis.

## 1. Introduction

From biomaterials, such as proteins and DNA in cells and viruses, to functional materials, represented by carbon nanotubes, fullerenes, and quantum dots, the analysis of nanomaterials has become increasingly important in medical, pharmaceutical, biochemical, and industrial fields [1,2,3,4,5]. The scale of materials to be analyzed is becoming smaller and smaller. Therefore, the importance of collection or manipulation techniques to handle nanomaterials is also increased [6,7,8].

**Figure 1:**
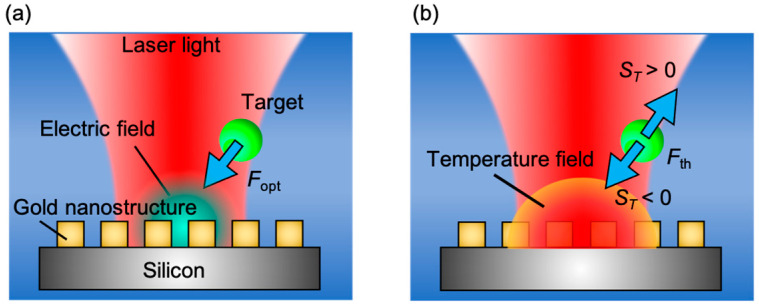
Schematic of manipulation of nanomaterials by (**a**) plasmon optical tweezers and (**b**) thermophoresis, induced around gold nanostructures. An optical force (**F**_opt_) acts on the target nanomaterials due to LSPR. When the heat generationis is predominant, by theremophoretic force (**F**_th_) moves nanomaterials away from the hot region when Soret coefficient is positive (*S_T_* > 0) whereas target materials migrate toward the hot region when Soret coefficient is negative (*S_T_* < 0).

Techniques, such as acoustic tweezers [9,10] or magnetophoresis [11,12], have often been used to manipulate various nanomaterials. In contrast to these techniques, we focused on optical techniques, such as plasmonic optical tweezers [13,14] and thermophoresis [15,16]. Here, we include thermophoresis as one of the photo-related phenomena, although it is not an optical method but a thermal one, because it can appear under optical irradiation due to photothermal conversion. These techniques have an advantage of controllable material size as small as a few nanometers compared with other techniques [17,18]. Especially, spatial resolution in the plasmonic method can be reduced to the size of the plasmonic nanostructures. Figure 1a shows the schematic of plasmonic optical tweezers. When a laser light with a certain wavelength is irradiated to gold nanostructures, the electrical field enhancement due to the localized surface plasmon resonance (LSPR) occurs by the resonance between the free electrons existing around the gold nanostructures and lasers. When tiny particles, target nanomaterials, exist around the gold nanostructures, optical radiation pressure is applied to the nanomaterials by the laser light irradiation. The optical radiation pressure **F**_opt_ is expressed by using electric field **E** as follows [13]:

(1) $$F_{\mathrm{opt}}\propto \;\nabla {\left|E\right|}^{2}$$

From Equation (1), the optical radiation pressure acting on the nanomaterials depends on the enhancement of the electric field. Additionally, because of the temperature gradient caused by the photothermal conversion, the thermophoretic force **F**_th_ in Equation (2) is also generated simultaneously on the nanomaterials [14].

(2) $$F_{\mathrm{th}}\propto \;S_{T}T\nabla T$$

where *S_T_* is the Soret coefficient, defined as the ratio of thermodiffusion coefficient to diffusion coefficient, and *T* is temperature. The direction of **F**_th_ is determined by *S_T_*, as shown in Figure 1b. When *S_T_* > 0, the nanomaterial moves from a high-temperature field to a low-temperature field. On the other hand, when *S_T_* < 0, the nanomaterial migrates in the reverse direction. Therefore, a detailed understanding of the temperature field around the gold nanostructures is quite important for advanced nanomaterial manipulation.

**Figure 2:**
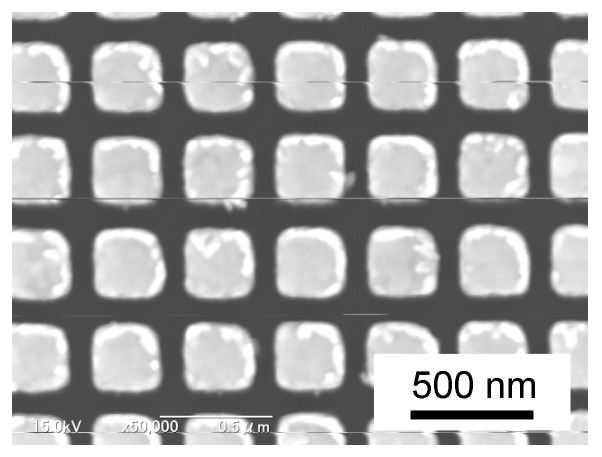
A SEM image of cuboid gold nanostructures fabricated by electron beam lithography.

The gold nanostructure can be fabricated by chemical modification [19] and electron beam lithography (EBL) [20]. In the present study, we employed EBL to fabricate gold nanostructures on a silicon substrate for the plasmonic nanomaterial manipulation. An example of the fabricated gold nanostructures is depicted in Figure 2. In the EBL-based nanostructure, the size, arrangement and gap of each gold island can be controlled. The effect of the gap has been investigated in some previous studies. Gap effect of gold dimers with two adjacent nanoparticles or structures was investigated by Su et al. [21]. They performed electromagnetic simulation with the gap from 5 to 30 nm. Yaraki et al. [22] investigated the electric field enhancement effect by irradiating plane waves into two silver nanostructures at different wavelengths and found that the electric field is most enhanced when the gap width between nanostructures was a few nm. When the electric field enhancement effect is large, the optical radiation pressure also becomes strong. Therefore, the gap width must be considered when fabricating gold nanostructures. However, it is difficult to fabricate structures with a gap width of a few nm by micro/nanofabrication. Hence, it is necessary to investigate the effect of electric field enhancement on the gap width that can be practically fabricated. Additionally, the thermophoretic force on the nanomaterials is also expected to change as the temperature field generated around the nanostructures varies by changing the gap width. Baffou et al. [23] investigated the effect of gap width on temperature field generation using obliquely irradiated plane waves on two 100-nm diameter spherical gold nanoparticles. Moreover, Pathak and Sarathi performed the numerical simulation of heat generation in gold nanodimer when irradiated at their LSPR [24]. However, plasmonic heat generation of gold nanostructures has not been discussed. Most of the previous studies only dealt with either of electric field enhancement and heat generation. In actual experiments, both phenomena simultaneously occur depending on the conditions. Our goal is to establish a way to find the optimal condition considering two concomitant phenomena. Therefore, this study focused on the gap width effect of cuboid gold nanostructures on both optical radiation pressure and thermophoretic force by numerical simulation to achieve effective manipulation of nanomaterials dispersed in liquid.

## 2. Numerical Simulation

### 2.1. Computational Model

**Figure 3:**
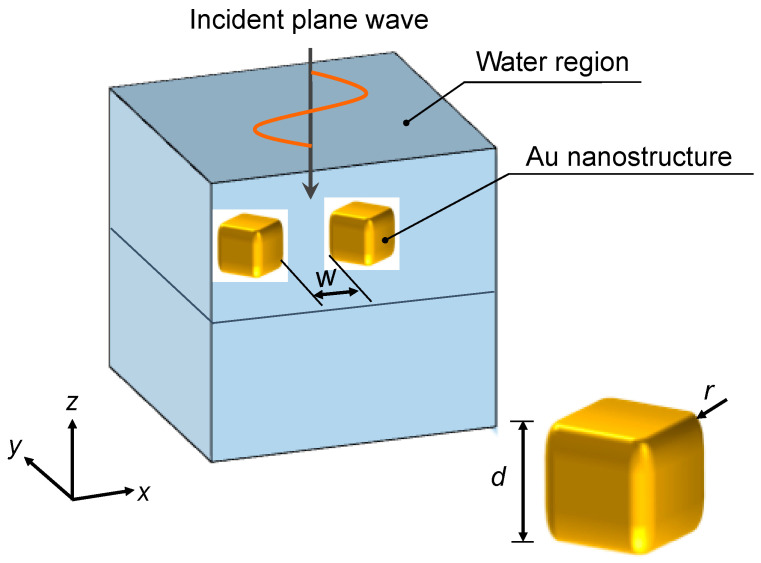
Numerical model of LSPR and photothermal calculation for gold nanostructures immsed in water with indicent plane wave from the top.

We utilized a finite-element software COMSOL Multiphysics for the numerical simulation in this study. Figure 3 shows a schematic of a three-dimensional simulation. The gold nanostructure has a cubic shape with a side length of *d* = 100 nm and its corners have a fillet radius *r* = 10 nm. The nanostructure is completely surrounded by water. Two gold nanostructures are aligned by edge-to-edge configuration to obtain a stronger interaction than face-to-face configuration in Figure 2. The parameters for simulation were gap spacing *w* and wavelength *λ* of an incident plane wave. Then, *w* and *λ* were changed in 10–100 nm and 400–1000 nm, respectively. The electric field **E** and temperature field *T* around gold nanostructures were sequentially evaluated by one-way coupling. Additionally, symmetric boundary conditions were applied to *xz* and *yz* cross-sections. Thus, a quarter model was used in the calculation.

### 2.2. Electric and Temperature Fields Calculation

The Helmholtz equation in the frequency domain analysis was employed as the governing equation to calculate the electric field **E** as follows:

(3) $$\nabla \times \left(\nabla \times E\right)-\frac{\omega }{c}\epsilon E=0$$

where *ω* is the angular frequency of the plane wave, *c* is light speed, and *ε* is the electric permittivity of the medium. In this calculation, a linearly polarized plane wave in the *x*-axis direction was given to the gold nanostructure as the incident electric field. The incident electric field of the plane wave is defined as follows.

(4) $$E_{x}=E_{0}\exp\left[-jk_{0}z\right]$$

where *E*_0_ is the amplitude of the plane wave’s electric field, and *k*_0_ is the wavenumber of the plane wave in vacuum. The value of *E*_0_ is calculated from the optical energy density *I* of the laser light beam by the following equation:

(5) $$E_{0}=\sqrt{\frac{2I}{n\varepsilon_{0}c_{0}}}$$

where *n* is the refractive index of the medium, *ε*_0_ is electric permittivity in vacuum, and *c*_0_ is the light speed in vacuum. Assuming that the laser beam has a circular cross-section and the energy density in the beam is uniform, the optical energy density *I* is simply calculated by the following equation.

(6) $$I=\frac{P_{\mathrm{in}}}{\pi {\left(\frac{d_{\mathrm{beam}}}{2}\right)}^{2}}$$

where *P*_in_ is the intensity of the laser beam and *d*_beam_ is the beam diameter of condensed light. In this study, we set *P*_in_ = 1 mW and *d*_beam_ = 3.4 µm, respectively, and *E*_0_ is determined from Equations (5) and (6) as *E*_0_ = 2.5 × 10^4^ V/m. The value of *d*_beam_ is determined by assuming the parameter of an objective lens having a magnification of 60×, which is typically used in experiments.

Subsequently, the steady-state heat diffusion equation was used as the governing equation to calculate the temperature field *T*. The governing equation is as follows:

(7) $$\lambda_{T}\nabla^{2}T+Q=0$$

where *λ_T_* is the thermal conductivity of water. The heat generation term *Q* corresponds to the heating by the electric field with the plasmon resonance around the nanostructures, determined by the following equation.

(8) Q=ω2ε0Im[εr]|E|2

where *ε_r_* is the electric permittivity of gold, which is defined by the Lorentz–Drude model [25].

### 2.3. Boundary Conditions

**Figure 4:**
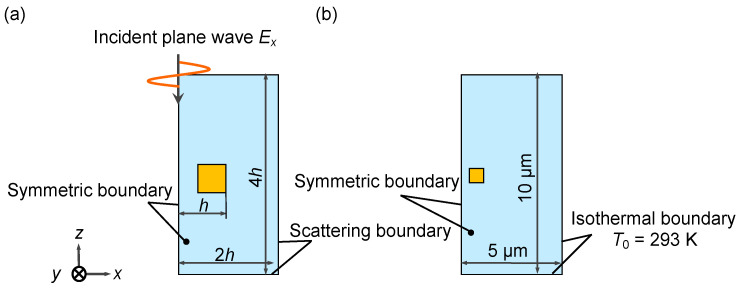
Boundary conditions for (**a**) electric and (**b**) temperature fields calculations.

The schematics of the boundary conditions for the calculation are shown in Figure 4. In the electric field calculations, a scattering boundary condition was set on the outer wall of the computational domain to prevent adverse effects due to the reflection of the electric field. The size of the domain is a cuboid with a height of 4*h* and a width and depth of 2*h* to prevent the influence of the computational domain’s size on the electric field generated by the gold nanostructure, where *h* is the distance from the center of the domain to the outer wall of the gold nanostructure as shown in Figure 4a.

Subsequently, the boundary conditions for the temperature field calculation are schematically shown in Figure 4b. An isothermal boundary condition of 293 K was adopted for the outer wall of the domain. The geometry of the temperature field calculation is a cuboid domain with a height of 10 µm and a width and depth of 5 µm, which is large enough considering the heat dissipation in water. We confirmed that number of the mesh elements in this simulation was sufficient as in Appendix A.

## 3. Results and Discussion

### 3.1. Electric Field Enhancement

**Figure 5:**
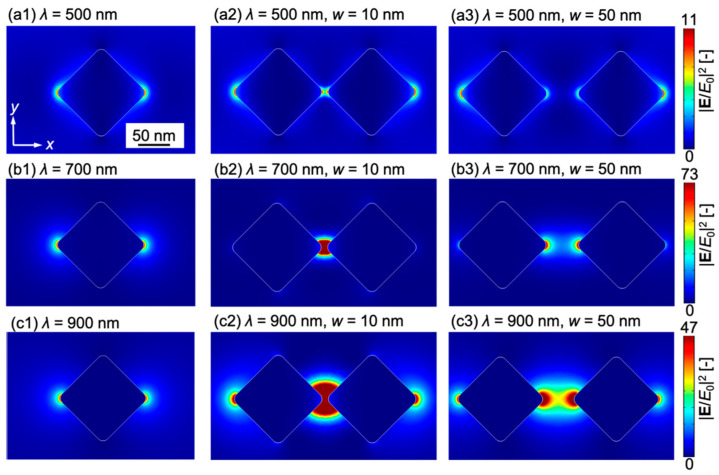
Electric field intensity |**E**/E_0_|^2^ in the *xy* cross section at the center of the structure: (**a1**) A gold nanostructure at wavelength *λ* = 500 nm, (**a2**) two gold nanostructures at *λ* = 500 nm, *w* = 10 nm and (**a3**) *λ* = 500 nm, *w* = 50 nm; (**b1**) A gold nanostructure at *λ* = 700 nm, (**b2**) two gold nanostructures at *λ* = 700 nm, *w* = 10 nm and (**b3**) *λ* = 700 nm, *w* = 50 nm; (**c1**) A gold nanostructure at *λ* = 900 nm, (**c2**) two gold nanostructures at *λ* = 900 nm, *w* = 10 nm and (**c3**) *λ* = 900 nm, *w* = 50 nm.

The enhancement of the electric field is evaluated using a parameter, enhancement intensity |**E**/*E*_0_|^2^, which is the electric field magnitude |**E**| acquired in the *x–y* cross-section at the center of the nanostructure normalized by the amplitude of the incident electric field *E*_0_. Figure 5(a1) shows the distribution of |**E**/*E*_0_|^2^ for a single gold nanostructure, and Figure 5(a2,a3) shows that for two gold nanostructures with *w* = 10 and 50 nm, respectively, at *λ* = 500 nm. Figure 5b,c also show |**E**/*E*_0_|^2^ for *λ* = 500 and 900 nm, respectively, for *w* = 10 and 50 nm.

The electric field is enhanced near the left and right edges of the gold nanostructures or between the gold nanostructures due to LSPR. Figure 5(a1–a3) shows that when *λ* = 500 nm, the electric field is enhanced to the same extent as that of a single structure for both gap widths *w*. On the other hand, Figure 5(b1–b3) show that for *λ* = 700 nm, a significant enhancement of the electric field occurs between structures at *w* = 10 nm, and at *w* = 50 nm, the electric field is also enhanced between structures, but only to the same extent as for an individual structure. Additionally, when *λ* = 900 nm, the large enhancement between gold nanostructures also appears at *w* = 10 nm, as depicted in Figure 5(c1–c3). These results indicate that the electric field enhancement increases with decreasing gap width, which is a similar trend to that obtained in a previous study [26].

**Figure 6:**
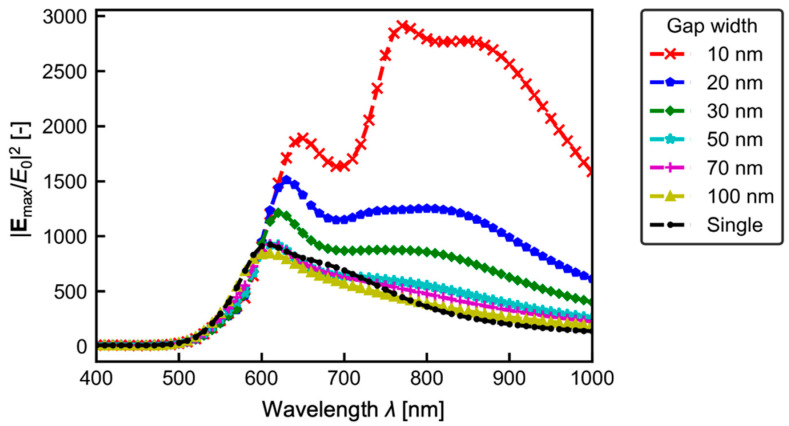
Maximum electric field enhancement in the numerical domain.

To further investigate the gap effect on the electric field, the relationship between the wavelength of the incident plane wave and the maximum electric field enhancement near the corners of the gold nanostructure in the computational domain |**E**_max_/*E*_0_|^2^ is shown in Figure 6. The results show that |**E**_max_/*E*_0_|^2^ has the first peaks at around *λ* ~ 600 nm for all gap widths. This peak wavelength shifts to the longer wavelength side as the gap width *w* decreases. As *w* decreases, the intensity of the electric field enhancement at around *λ* ~ 800 nm gradually increases, and at *w* = 50 nm, the enhancement is almost the same as the first peak, and at *w* = 10 nm, the value becomes maximum. These results are considered because when gold nanostructures become closer to each other, a state of polarization is generated inside the gold nanostructures by LSPR, and a strong electric field is induced by the interaction of the structures with each other. Yaraki et al. [22] showed that polarization states, such as dipoles and quadrupoles inside the structure, change with incident wavelength, resulting in the interaction between the two structures and changes in the intensity of the electric field. They have also confirmed that two peaks of electric field enhancement are generated. The two peaks are also observed in the distribution of *w* = 10 nm in Figure 6, suggesting a similar phenomenon in our calculation.

Considering the optical force on nanomaterials existing around the gold nanostructures, the optical radiation pressure increases due to the enhanced electric field around the nanostructures. The present calculation results shown in Figure 6 indicate that the variation |**E**_max_/*E*_0_|^2^ compared with a single nanostructure is within twice when *λ* ~ 600 nm and that for *λ* ~ 800 nm reaches approximately 10. This fact would suggest that narrower gap always provides a beneficial effect on nanomaterial manipulation. The irradiation light wavelength needs to be selected as near infrared light should be used when the gap width can be less than 20 nm, and if not, red light should be used. In practical application, the firm fabrication of the narrow gap less than 20 nm is quite difficult and might cause an increase of costs. Therefore, one carefully needs to find the optimal gap for application regarding the manipulation performance or production cost.

### 3.2. Temperature Field

**Figure 7:**
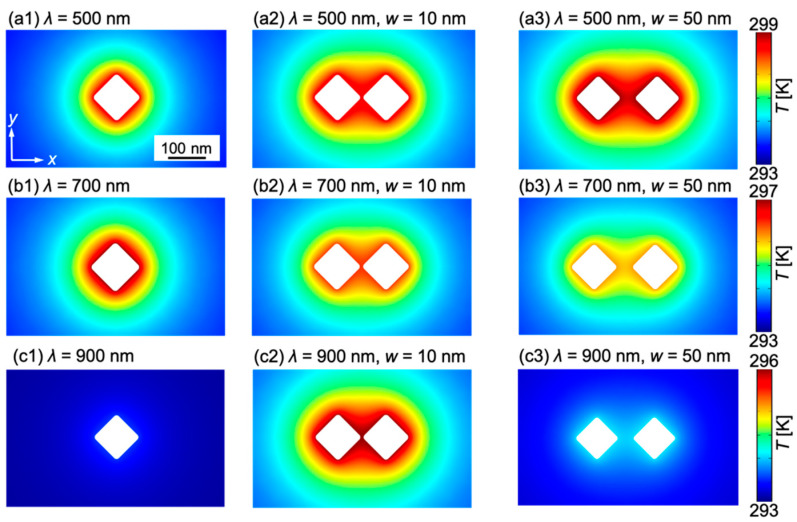
Temperature field *T* in *xy* cross section at the center of the structure: (**a1**) A gold nanostructure at *λ* = 500 nm, (**a2**) two gold nanostructures at *λ* = 500 nm, *w* = 10 nm and (**a3**) *λ* = 500 nm, *w* = 50 nm. (**b1**) A gold nanostructure at *λ* = 700 nm, (**b2**) two gold nanostructures at *λ* = 700 nm, *w* = 10 nm and (**b3**) *λ* = 700 nm, *w* = 50 nm; (**c1**) A gold nanostructure at *λ* = 900 nm, (**c2**) two gold nanostructures at *λ* = 900 nm, *w* = 10 nm and (**c3**) *λ* = 900 nm, *w* = 50 nm.

The temperature field *T* acquired in the *x–y* cross-section at the center of the structure is shown in Figure 7. Figure 7(a1–a3) shows the temperature distributions obtained at *λ* = 500 nm for a single structure and two structures having *w* = 10 and 50 nm, respectively. Figure 7b,c show the distributions obtained at *λ* = 700 and 900 nm, respectively, for *w* = 10 (Figure 7(b2,c2)) and 50 nm (Figure 7(b3,c3)).

Figure 7 shows that when there is only a single nanostructure, the isotropic temperature field is generated around the nanostructure. From Figure 7(a1–a3), at *λ* = 500 nm, the maximum temperature field was obtained when two nanostructures with *w* = 50 nm were used. In Figure 7(b1–b3), at *λ* = 700 nm, the largest temperature distribution was obtained when a single nanostructure was used, and the temperature rise was larger for *w* = 10 nm than for *w* = 50 nm when two structures were used. Figure 7(c1–c3) shows that at *λ* = 900 nm, the temperature is largest when *w* = 10 nm between two nanostructures. These results indicate that the effect of the gap width on heat generation is very sensitive to the wavelength of the incident light.

**Figure 8:**
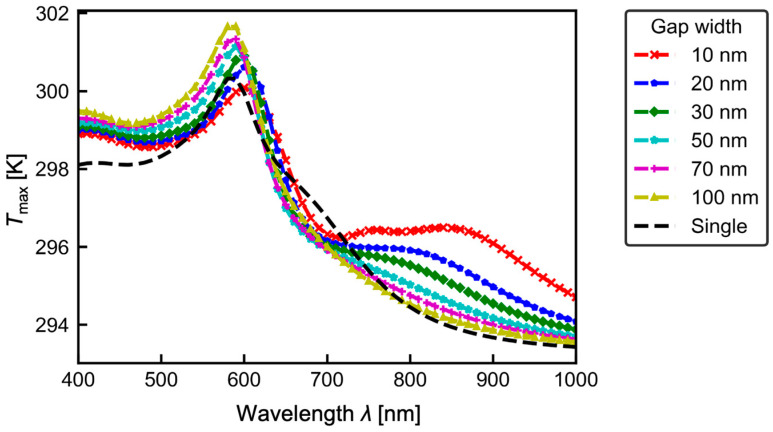
Maximum temperature *T*_max_ in the numerical domain.

**Figure 9:**
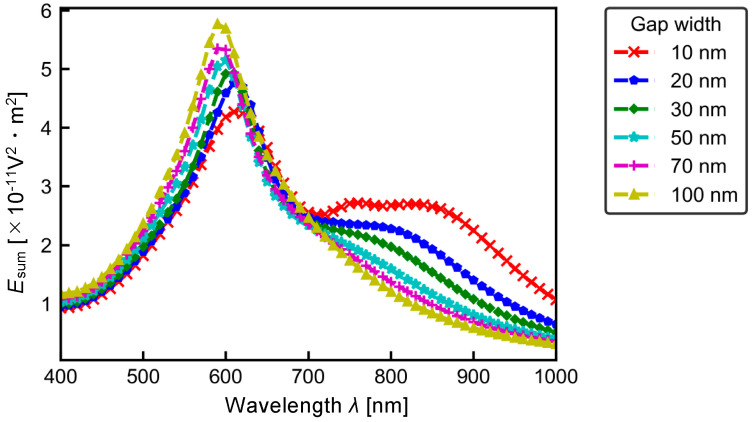
Volume integral of |**E|**^2^, *E*_sum_, inside the gold nanostructures.

Subsequently, to investigate the effect of the gap width on the temperature at each wavelength, the relationship between the wavelength of the incident plane wave and the maximum temperature *T*_max_ was examined. The results are shown in Figure 8. In all cases, the location where *T*_max_ appeared was at the surface of the gold nanostructures. From Figure 8, *T*_max_ shows a peak at *λ* ~ 600 nm for all gap widths. Below the peak wavelength of *λ* ~ 600 nm, the temperature is higher for larger gap widths. Moreover, at *λ* ~ 850 nm, the temperature is higher for smaller gap widths. This is due to the influence of the electric field inside the gold nanostructures because Equation (6) is set to the heat generation term. Figure 9 shows the relationship between the wavelength of the incident plane wave and the volume integral of the electric field |**E|**^2^ inside the gold nanostructure, i.e., *E*_sum_ defined by Equation (9).

(9) $$E_{\mathrm{sum}}=\int_{\mathrm{Au}}^{}{\left|E_{\mathrm{Au}}\right|}^{2}dV$$

From Figure 9, the value of *E*_sum_ has a sharp peak at *λ* ~ 600 nm with positive dependence of the gap width and broad peak at *λ* ~ 850 nm with negative dependence of the gap width. These trends are similar to those in *T*_max_ shown in Figure 8. Then, this result indicates that the generation of the temperature field is due to the contribution of the electric field inside the gold nanostructure. Moreover, the difference between Figure 8 and Figure 9 can be found whatever the wavelength region. This is attributed to the wavelength dependent energy as in Equation (8); light with short wavelength has higher energy.

Next, the influence of gap width on thermophoretic manipulation performance for nanomaterials is discussed. A temperature rise generally induces thermophoretic force for the nanomaterials. However, the variation of *T*_max_ depending on the wavelength is within several K. This difference results in the difference of the thermophoretic force about 1% because the thermophoretic force (Equation (2)) is proportional to the absolute temperature *T*. Therefore, it can be said that the effect of the gap width change on the thermophoretic force is considered negligibly small. Consequently, we can state that one does not need to take primary care of the gap, unlike with the plasmonic tweezers.

## 4. Conclusions

This study numerically investigates the effects of gap width on the electric and temperature fields generated around gold nanostructures when irradiating a plane wave that assumes a laser light beam. The electric field calculations show that as the gap width *w* decreases, the electric field is gradually enhanced at wavelengths both around 600 nm (red) and 800 nm (near infrared), reaching the maximum at *w* = 10 nm. Especially, the near infrared peak becomes dominant when *w* < 20 nm. The temperature field calculation shows that the plasmonic heat generation around gold nanostructures has complicated the dependence of the gap width depending on the wavelength of incident waves.

The effect of gap width on the migration performance for nanomaterials existing around the gold nanostructures is expected to be greatly different depending on the manipulation principle. The optical radiation pressure is sensitive to the gap width due to the large change in the electric field enhancement. On the other hand, there is no significant difference in the thermophoretic force, although the temperature rise shows some trends that depend on the gap width, suggesting that the effect of the gap width on the thermophoretic power is small.

## Figures and Tables

**Figure A1:**
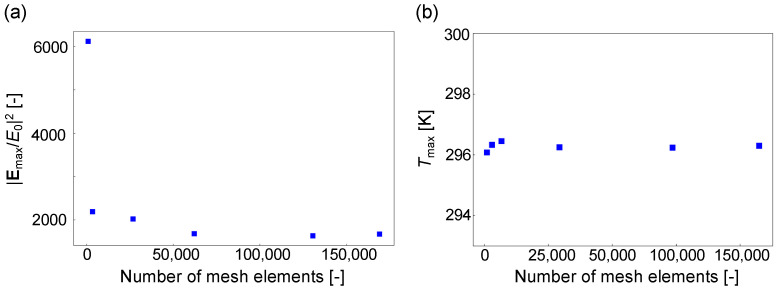
Mesh dependence of (**a**) maximum electric field enhancement and (**b**) maximum temperature.

## Data Availability

The data presented herein are available upon request from the corre-sponding author.

## Appendix A

Mesh number dependence in electric and temperature field calculations is presented in Figure 7. Figure 7a,b shows the relationship between the number of mesh elements and the maximum electric field enhancement |**E**_max_/*E*_0_|^2^ and the maximum temperature *T*_max_, respectively. Here, the results when *λ* = 700 nm and *w* = 10 nm are shown. The electric field converges when the number of mesh elements is over 50,000 (Figure A1), whereas the thermal calculation does not indicate the mesh number dependence. Therefore, we employed the mesh numbers of 62,000 and 30,000 in the electric field and thermal calculation, respectively.
